# Late translational research: putting forward a new model for developing new anti‐cancer treatments that addresses the needs of patients and society

**DOI:** 10.1002/1878-0261.12431

**Published:** 2019-01-19

**Authors:** Denis Lacombe, Jan Bogaerts, Bertrand Tombal, François Maignen, Leeza Osipienko, Richard Sullivan, Vassilis Golfinopoulos

**Affiliations:** ^1^ EORTC Headquarters Brussels Belgium; ^2^ Cliniques Universitaires Saint‐Luc Université Catholique de Louvain Brussels Belgium; ^3^ NICE Scientific Advice National Institute for Health and Care Excellence London UK; ^4^ Institute of Cancer Policy, Conflict and Health Research Group King's College London & Kings Health Partners Comprehensive Cancer Centre London, UK

**Keywords:** cancer clinical research, effectiveness, healthcare, patient centredness, translational research

## Abstract

Bringing therapeutic innovation and the latest science to routine patient care, while safeguarding principles of affordability and equality, is a challenging mission in the current complex multi‐stakeholder environment. Precision oncology and new approaches to clinical trials (methods and clinical setting) have dramatically changed clinical research and the clinical development of new treatments. Improved understanding of molecular biology and immunology paves the way for innovative pharmacological approaches. However, we argue that the evidence generated during the clinical development of these new products for the purpose of obtaining marketing authorisations often does not address fundamental questions concerning the impact of these new interventions on the most relevant clinical outcomes: namely, quality of life and patient survival. Similarly, patient populations (for example defined by biomarkers), treatment duration, and sequence and combination of treatments within current treatment pathways are often poorly defined by clinical developments for regulatory purposes. Finally, the lack of integrated translational research within the pathway of development is a major limiting factor to delivering cost‐effective and affordable, evidence‐based care to clinical practice. This leaves many gaps in the knowledge on the efficacy and therapeutic use of medicines, which can impose a significant financial burden on healthcare systems, possibly to the detriment of more cost‐effective interventions. We argue that policy changes are required to integrate clinical research and healthcare to inform clinical practice. New routes toward optimising the integration of drug development and care are being proposed to achieve this ultimate goal.

**Abbreviations**
CDF: cancer drugs fund
EORTC: European Organisation for Research and Treatment of Cancer
FDA: Federal Drug Administration
HTA: health technology assessment
NICE: National Institute for Health and Care Excellence
PFS: progression‐free survival

## 1 Introduction: hope and uncertainties in cancer clinical research

**Figure 1:**
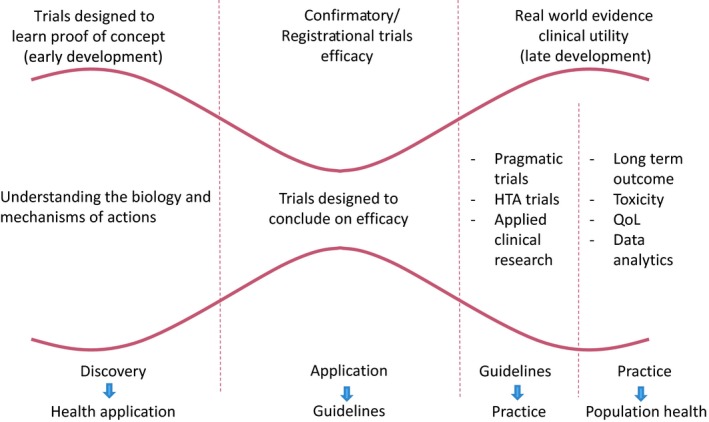
A revisited framework of clinical development in clinical practice. Patient‐centred approaches plead for the right and left part of the figure to fall under the expertise of independent stakeholders.

Understanding the tumour and host biology is critical to tailoring treatment and is today referred to as what we define as the research foundation to precision oncology. Translational research builds on basic science for potential clinical impact and conversely to understand clinical observations through the documentation of biology. Translational research should sustain the process from early into late clinical research to document fully the use of treatments in clinical practice. In recent years, rapid knowledge of molecular characterisation of tumours as well as a growing understanding of the tumour microenvironment and, more specifically, immuno‐oncology have profoundly changed therapeutic strategies in oncology (Emens *et al*., 2017; Lyman and Moses, 2016; Shrager and Tenenbaum, 2014). Despite significant therapeutic progress in some cancers, e.g. melanoma (Su and Fisher, 2016), there are many limitations to the implementation and optimal use of new treatments derived from precision oncology (Chin, 2015; Hughes, 2017; Tannock and Hickman, 2016). Applied clinical research is part of late clinical research and focuses more specifically on documenting the appropriate use of treatments in clinical practice (Fig. 1). It typically builds on clinically relevant end‐points to answer questions which are central to patient treatment and management. Therefore, it builds on clinical trials which are being referred to as pragmatic trials, usually targeting broader patient populations. This part of clinical research is therefore intuitively fit for purpose for informing health technology assessment bodies (HTA) on implementation of therapeutic strategies in healthcare.

The alarming low therapeutic benefit of many new anti‐cancer medicines derived from the precision oncology approach, has been extensively discussed (Davis *et al*., 2017; Prasad, 2017). In addition, cut‐off values for biomarkers, as well as patient populations, are often ill defined, as well as the understanding of the patterns of resistance or the nature and timing of escape mechanisms. Optimisation of the sequential use of therapeutic agents and/or combinations are rarely addressed in drug development (Lacombe *et al*., 2017). Further complexity arises from the lack of unbiased independent head‐to‐head relative effectiveness trials of anti‐cancer agents, further adding to the uncertainty in translating evidence from the regulatory clinical development to the real world (Flacco *et al*., 2015). All this converges on healthcare systems, which are increasingly ‘expected’ to take up new anti‐cancer medicines with consistently increasing uncertainty as to their actual therapeutic benefit and their place in increasingly complex models of care.

Appropriate patient‐centred approaches where translational research is central to the understanding of these gaps are crucial to address these deficiencies (Biankin *et al*., 2015; Roberts *et al*., 2012). HTA bodies involved in the reimbursement decisions of new technologies and publication of clinical guidance also face very similar issues. We therefore argue that the sequential integration of translational and clinical research needs to be strengthened and reorganised in the common interest of clinicians, sponsors, HTA bodies and, mostly importantly, patients, using a longitudinal approach of the follow up of patients throughout the course of their disease.

## 2 Clinical and translational research should pave the way to regulatory sciences

The technological revolution has led to new types of clinical trials, generating different and multidimensional datasets that require novel bio‐informatics solutions for appropriate interpretation. However, clinical research still uses classical approaches in the development of a protocol, for a specific drug addressing a certain target, and therefore searching for relatively rare subsets of patients who may benefit from the intervention. Moving into patient‐centred clinical research requires the implementation of integrated and collaborative molecular screening platforms, integrating curated biologically annotated clinical data (Meric‐Bernstam *et al*., 2015; Trusheim *et al*., 2016). Screening platforms can provide solutions to bring translational research to patients not only at a certain point in time for access to a specific matched treatment, but also over time as the disease evolves, for optimal subsequent treatment decisions.

This speaks to the need for a new paradigm in clinical and translational research as activities from early development to pragmatic research. A transition to pragmatic applied clinical and translational research needs access to a much wider, more real‐world‐relevant community of patients. It also requires that clinicians and cancer researchers at community centres be trained in clinical research, which remains poorly supported in many countries in Europe. Biopsy/re‐biopsy, even if not yet the rule, is becoming more feasible as the invasiveness of technologies decreases and/or is supported, if not replaced, by powerful imaging solutions and/or liquid biopsies which could rapidly become a solution for patient surveillance. These advances should rapidly allow the performance of pragmatic trials through the access to biological materials in large networks. An organised large network with sustainable infrastructures that can optimise such trials is needed at the European level. The European Organisation for Research and Treatment of Cancer (EORTC) has recently extended its capabilities in this direction, and today represents a unique European solution for the implementation of precision oncology in Europe, which could be timely at a point when Europe looks into new solutions such as the cancer mission (Celis and Pavalkis, 2017). The EORTC SPECTA platform for addressing precision oncology, launched in 2014 (Lacombe *et al*., 2014), has been an incubator for learning how to organise science, operations, and clinical and regulatory requirements in a large, international, all‐inclusive way which can now serve many partners and projects such as EURACAN, the European Reference Network for rare adult cancers (Girard, 2018). SPECTA is today supporting the translational research component of EORTC studies, providing quality assurance for the adequate process of translational research alongside the validation of clinical hypothesis.

There is a need for systematic and specific access to biological specimens in a structured manner and in a constant process of adaptation as science evolves to tailor treatments. Most importantly, experience shows that the underlying biological mechanisms cannot alone inform clinical practice and we argue that clinical end‐points are still necessary to inform the value of new anti‐cancer products.

## 3 The current limitations of clinical development in oncology

A four‐step process, from basic scientific discoveries to ‘candidate applications’, from ‘candidate applications’ to proven interventions, from proven interventions to clinical practice, and from clinical practice to population health impact, has been suggested as a conceptual framework (Khoury *et al*., 2010). Moving from the early stages of drug development into clinical practice is recognised as being a continuum in which each step must optimally prepare for the next one. This conflicts with development plans targeting accelerated approvals, which are frequently based on surrogate end‐points and limited populations, and thus do not address the aim of proven interventions, creating a paradigm where such proof may not be established in the post‐approval setting. The process of drug development is often interrupted in its early stage when such critical information is not immediately foreseeable (Gellad and Kesselheim, 2017). Moreover, when medicines are approved on the basis of surrogate end‐points, only a fraction of these products demonstrate improvements in survival or quality of life during the post‐authorisation commitments (Davis *et al*., 2017; Kim and Prasad, 2015).

By definition, registrational clinical trials in oncology are designed to fulfil regulatory requirements (i.e. demonstrate that a new medicine has a positive risk/benefit balance) and are not necessarily designed to inform clinical practice. This precludes research by allowing access to new therapies which become part of the ‘standards of care’ before a firm empirical base can be established (Light and Lexchin, 2015a,b).

Registrational trials are often designed to minimise any biases or remove external factors which could interfere with the efficacy of the new therapy, consequently these studies are conducted on selected patient populations (generally healthier than the patients treated in clinical practice), using primary end‐points of debatable clinical relevance [progression‐free survival (PFS)]. There is also a substantial overlap between orphan designations and oncology medicines; on that basis, new oncology therapies are developed using trial designs which can significantly bias the estimate of the treatment effect (e.g. single‐arm trials, interim analyses or crossover of patients once the primary objective on a surrogate end‐point such as PFS is achieved before the evidence of efficacy on overall survival is observed). Such studies rarely contain patient‐centred outcomes such as self‐reported quality of life using generic scales (e.g. EQ‐5D). There is a fundamental need to keep in mind at all times that the final objective of clinical research is to bring added value to patients. New technologies need new comprehensive and multidimensional criteria agreeable to all stakeholders to impact on clinical practice in a meaningful manner and subsequently make a clear difference for healthcare and society. The generalisability of the trial results to clinical practice is further impaired by a poor choice of comparators (if the trial is conducted with a control arm) that do not represent current medical practice (Tao and Prasad, 2018). The poor external validity of these studies further complicates matters by adding to the uncertainty around how to use treatments. It is often left to HTA bodies to reconcile regulatory and clinical practice requirements, but this only partially addresses the issue, as currently it is not clear whether Health Technology advice is incorporated into registrational trials, and these trials still remain very limited in terms of time, size, and composition to answer relevant clinical questions. In addition, regulatory trials frequently address selected tumour types that are of commercial interest, whereas rarer indications that may be economically non‐viable are often not the subject of significant development of new treatments. Therefore, there is an obvious gap between early and late drug development which is often artificially split by the process of marketing authorisation, after the granting of which the commercial sector may no longer pursue research for treatment optimisation, or indeed may not be able to do so as the authorised drug that is considered standard of care crowds out any future research.

Applied clinical research is needed to define effective practice‐changing approaches, to bring therapeutic innovation efficiently and consistently to cancer patients, and to address patient populations who do not fall within the primary interests of the commercial sector. Historically, independent researchers and networks have undertaken such a research agenda. The EORTC is one of the leading pan‐European examples of such a network that has changed practice for many cancer types through patient‐oriented clinical research. Applied clinical and translational research can take into account the HTA dimension of therapeutic interventions, focusing on patient‐centred end‐points, and complementing the regulatory dimension of drug development. Applied clinical research is essential for informing real‐life implementation and can also support continued and structured research in healthcare systems, delivering useful progress that is relevant to patients (Ioannidis, 2016). In this context, it is also important to point out academic initiatives in oncology addressing clinically meaningful benefit and value of new cancer treatments, such as those driven by the American Society of Clinical Oncology and the European Society for Medical Oncology. Although they add a new dimension to this area, they may not take into account the full environment and the continuum of information that needs to be developed to make good clinical decisions. Such initiatives help to identify the magnitude of the issues we are faced with, but their societal impact remains to be demonstrated (Cherny *et al*., 2015; Schnipper and Schilsky, 2017; Schnipper *et al*., 2015). As a consequence, the impact of recently approved new drugs has also been under scrutiny (Davis *et al*., 2017). Overall, the evidence‐based net efficacy of today's anti‐cancer development paradigm has been judged by many to be marginal (Prasad, 2017, 2018). This clearly emphasizes the need to establish a structured process to address the serious and growing schism between regulatory pathways for drug development and the real‐world needs of patients, clinicians, and societies.

Furthermore, pharmaceutical companies are in a situation of conflict of interest when it comes to optimising the development of biomarker and refining patient selection, as these investigations will ineluctably lead to a restriction in the target population, and therefore in the market. We illustrate these issues with a specific example here. Trials addressing immunotherapy and more specifically check‐ point inhibitors have changed the approach to treating melanoma. However, the clinical development of these products did not adequately inform the optimum treatment duration, which is a critical issue for clinical practice. The DANTE trial in the UK and the STOP‐GAP trial in Canada, both supported by independent funders, are applied research clinical studies which were specifically designed to address this crucial question after the authorisation of these products [Duration of Anti‐PD‐1 Therapy in Metastatic Melanoma (STOP‐GAP)—Full Text View—ClinicalTrials.gov, (2016); ISRCTN—ISRCTN15837212: DANTE: Duration of Anti‐PD1 therapy for Melanoma (2018)].

Recommendations to qualify and validate biomarkers as well as establish their clinical utility in the context of cancer drug development have been proposed by multi‐stakeholder groups such as EORTC, AACR, and EMA (Salgado *et al*., 2017). Such multi‐stakeholder analysis has revealed a rapidly changing environment where regulatory science, drug development, and data‐driven healthcare systems need realignment and convergence. The concept for approaching anti‐cancer treatment development through research platforms that optimise discoveries in biology, building on translational research through the entire process until optimal access to treatments for patients in routine practice, has been posited (Burock *et al*., 2013; Lacombe *et al*., 2014). While early stage drug development should be based on strong biological evidence documenting tumour biology and mechanism of action, late clinical and translational research should aim at optimising appropriate and rational implementation in clinical practice and directing it towards real‐world patient management. It has been postulated that late‐stage, pragmatic, applied clinical and translational research might become an integral part of patient treatments, structuring the use of new agents in healthcare based on robust medical evidence that would provide intelligence to patients, doctors, and policymakers to establish value (through rational price setting) and clinical practice guidelines (Lieu and Platt, 2017). Such research in oncology care settings can be implemented in large networks with decentralised investigational sites, reflecting the real world as opposed to the drug development ‘world’. This step is not currently part of the routes leading to treatment access or treatment commissioning in healthcare settings. An example that addresses this need is the cancer drugs fund (CDF) within the National Institute for Health and Care Excellence (NICE) in the UK. The CDF can be used when the committee considers plausible a potential for a drug to satisfy the criteria for routine commissioning, but where significant clinical uncertainty remains. NICE aims to make a positive or negative recommendation for use of a treatment in the National Health System. However, should the evidence for cost‐effectiveness be plausible but insufficient, the NICE appraisals committee has the option to refer oncology products for entry to a temporary coverage arrangement via the CDF. Under the CDF, additional data need to be generated over a 2‐year period to inform the review of the new treatment either to recommend it for routine clinical practice or not. All CDF appraisals will follow the standard technology appraisal process, with amendments relating to the CDF described in PMG19 Addendum A (NICE 2016).

If well structured, an appropriate balance between pre‐authorisation interventional trials, trials for robust evidence for clinical practice, and post‐authorisation morbidity–mortality studies and surveillance for continued knowledge development through population cohorts could also be a way forward (Bayer and Galea, 2015; Choudhry, 2017; Fiore and Lavori, 2016; Ford and Norrie, 2016; Frieden, 2017; Sherman *et al*., 2016).

As it stands, solutions which claim that the real world should be an expanded experimental population to validate early clinical access are contrary to the fundamental need to establish real value for innovations in societies and simply add to issues of waste in healthcare systems (Chalmers *et al*., 2014; Chan *et al*., 2014; Glasziou *et al*., 2014; Ioannidis *et al*., 2014; Salman *et al*., 2014). For that reason, we argue that there is a now compelling need to ensure that independent, patient‐centred applied research is embedded in the development of new treatments at the time of their authorization, taking precedence as new treatments are implemented in the healthcare setting. This proposal would have major advantages. First, there would not be any missed opportunities for patients, as they would benefit from new technologies. Secondly, this organised applied research could be specifically designed to collect the crucial evidence described above (e.g. evidence on survival gains). Thirdly, this research could be included in regulatory post‐authorisation commitments, and HTA‐managed entry agreements and risk‐sharing schemes designed to address clinical uncertainty. The funding of such research could be provided by risk‐sharing agreements between healthcare systems (as part of the treatment of new patients) and companies. Finally, we believe that new technologies would facilitate the collection and analysis of data and would minimise the additional burden for the healthcare systems (and the patients, e.g. for the collection of quality of life information). In conclusion, a fundamental way of delivering the required empirical base for equitable and affordable cancer care (note: for not only anti‐cancer medicines but all other new technologies) would be to realign clinical and translational research across stakeholders. It should be noted (even if this is not the primary objective of our article) that this principle goes beyond the field of oncology and should be applied across all therapeutic areas.

For the reasons mentioned above, we plead that the utility of therapeutic interventions for societies must be radically improved without any loss of (real) opportunity for patients. It has been suggested that big data approaches in population‐based observational national and pan‐national cohorts would help us to understand the use and impact on outcomes of treatments in the real world. This is not without challenges but the opportunities to utilise these approaches need to be understood. The optimal balance between different methods for understanding of the true value of treatments still needs to be evaluated, but it must not compromise the robustness of the conclusions on which treatment decisions are based (Booth and Tannock, 2014). An iterative approach to clinical and translational research to accompany the continuum of research and healthcare is warranted to address new and emerging treatment solutions and new technologies, not just limited to drugs but also including specific approaches such as particle therapy or robotic surgery, and the integration of all treatment modalities into care. Improved certainty can only be delivered by prospective, structured clinical research programmes. While randomisation should remain the gold default standard, it is indisputable that in specific situations, it may not always be feasible. Prospective registries may bring in substantial added value to specific clinical situations through systematic data structuring supporting decision‐making. However, uncertainty is inevitably higher in such setting. Integrating the role of registries and any systematic prospective data collection are part of the continuum from research to healthcare. A clear understanding of the limitations this may have when changing practice, is required. The clinical utility of therapeutic interventions needs a high level of evidence. Relaxing methodology for effectiveness requires careful assessment, and research on new methodologies in this area is needed. Registries intended for societal decision must follow criteria which do not differ dramatically from those for clinical trials. The only reference document for the use of real‐world data issued by the Federal Drug Administration (FDA), though for medical devices, points in that direction (U.S. Department of Health and Human Services *et al*., 2016). Registries are not necessarily easier to set up or conduct. They deliver potentially less robust evidence for society and should be considered in specific situations, for example when equipoise is not ensured.

## 4 The way forward: the need for an integrated vision of patient centredness

Cancer clinical research within the context of healthcare systems is overdue for transformation. To become a paradigm with real impact, equality, affordability, and high quality utilisation of new treatments need intimate cross‐links between research and care for precision oncology. We also need to recognise that precision oncology is only one part of the solution in the fight against cancer. Therapeutic progress across all the fronts is beyond the remit of any single organisation, company or stakeholder. Current approaches to drug development are becoming obsolete and healthcare spending unsustainable. Re‐engineering the forms and methods of therapeutic innovation around the understanding of biology, and implementing solutions for documenting the spatial and temporal heterogeneity of tumours, through collaborative programmes helping to organize and sort groups of diseases, would be of benefit to all, facilitating access for patients to clinical research and beyond. But this needs to be integrated with earlier and more in‐depth socio‐economic studies to provide the policy context for individual countries. Indeed, clinical research has reported decreasing activity and efficacy of new treatments as development moves from early to late stages (Hemkens *et al*., 2016; Nagendran *et al*., 2016). Thus, true effectiveness in healthcare settings has rarely been documented. At a time when Europe is addressing fundamental concepts in the fight against cancer through a mission‐oriented approach (Celis and Pavalkis, 2017), a rational long‐term vision must be established that establishes deeper empirical foundations for the real world. We propose to revisit the models of clinical development to embrace an approach which will optimise the expertise of the various stakeholders. The proposed solution as depicted in Fig. 1, would structure clinical development in three major, possibly overlapping, sections. Independent patient‐centred programmes should inform patients on the biological characteristics of their diseases, and then optimise their access to matching treatments. Applied clinical research programs would confirm the value of treatments in clinical practice, providing the grounds for long‐term follow‐up outcome research programmes. This could optimally be achieved by revisiting and balancing the role of stakeholders alongside the process. As previously discussed, this could result in an optimised framework for managed access agreements (risk‐sharing).

Building from existing and developing solutions such as the Comprehensive Cancer Centers, which, if and when further deployed, will make implementations of technologies such as liquid biopsies possible in larger patient populations. Supported by developing capacities in information technology, decentralised policy, enabling comparative effectiveness in healthcare (Table 1), reaching all patients could become a reality. Medical reversal is the consequence of accepting new standards of treatment based on low evidence. It raises false hope for patients and has a subsequent cost to society (DeLoughery and Prasad, 2018; Prasad, 2018; Prasad and Cifu, 2011). Reversal represents a suboptimal research agenda where interventions are deployed in populations, often for decades, before fundamental studies are performed to evaluate efficacy. While research is responsible for delivering new therapeutic options based on the highest and most reliable standards of methodology, society is responsible for ensuring access to treatments in an equitable and affordable manner. The time has come to revisit the entire business model. The setting up of the European mission is, we hope, the place where this should happen for the sake of all European cancer patients.

**Table 1:** Features to consider in implementing translational research in applied clinical research for data‐driven healthcare in the context of the mission‐oriented approach to cancer

1.	Adapt the process of clinical research for precision oncology to inform clinical practice
2.	Realign the sequence and roles of stakeholders in the process of clinical research, embedding applied clinical research
3.	Implement solutions from research into healthcare and back, through optimal coordination of all stakeholders
4.	Evolve towards patient centredness addressing knowledge development and changing practice
5.	Align the needs of patients and doctors with societal priorities for access to innovation
6.	Establish forward‐looking and flexible systems reaching all patients as technology evolves
7.	Implement pragmatic solutions associating and educating all healthcare providers in the delivery of constantly evolving standards of care according to latest science

## Conflict of interest

The authors declare no conflict of interest.

## Author contributions

All authors have contributed to the concept, writing, and reviewing of this paper.
